# Evaluation of the epidemiologic, clinical, radiologic, and treatment methods of patients with subacute and chronic meningitis

**DOI:** 10.1186/s12883-022-02873-1

**Published:** 2022-09-10

**Authors:** Niloufar Bineshfar, Ali Rezaei, Alireza Mirahmadi, Shervin Shokouhi, Farid Javandoust Gharehbagh, Mehrdad Haghighi, Ali Amini Harandi, Maziar Shojaei, Mahtab Ramezani, Anahita Zoghi, Kourosh Gharagozli, Legha Lotfollahi, Ilad Alavi Darazam

**Affiliations:** 1 School of Medicine, Shahid Beheshti University of Medical Sciences, Tehran, Iran; 2 Infectious Diseases and Tropical Medicine Research Center, Shahid Beheshti University of Medical Sciences, Tehran, Iran; 3 Department of Infectious Diseases and Tropical Medicine, Loghman Hakim Hospital, Shahid Beheshti University of Medical Sciences, Makhsoos St, South Kargar Ave, 1333635445 Tehran, Iran; 4 Brain Mapping Research Center, Shahid Beheshti University of Medical Sciences, Tehran, Iran; 5 Department of Neurology, Loghman Hakim Hospital, Shahid Beheshti University of Medical Sciences, Tehran, Iran; 6 Skull Base Research Center, Shahid Beheshti University of Medical Sciences, Tehran, Iran; 7 Department of Nephrology, Loghman Hakim Hospital, Shahid Beheshti University of Medical Sciences, Tehran, Iran

**Keywords:** Chronic meningitis, Subacute meningitis, CNS infection, Tuberculosis, Epidemiology

## Abstract

**Background:**

Meningitis is known as a meningeal inflammation accompanied by pleocytosis in the cerebrospinal fluid (CSF), and can be classified into acute, subacute, and chronic meningitis based on symptoms duration of ≤ 5 days, ≥ 5 days and ≥ 4 weeks, respectively. Subacute and chronic meningitis are caused mainly by indolent infectious agents and noninfectious causes such as autoimmune, and neoplastic. In this study, we investigated the characteristics, diagnosis, and treatment of subacute and chronic meningitis.

**Methods:**

We extracted the medical records of patients with chronic and subacute meningitis who were referred to three tertiary centers from Jun 2011 to Jun 2021. Initially, 2050 cases of meningitis were screened, and then 79 patients were included in the study.

**Results:**

Headache (87.3%), nausea and vomiting (74.7%), fever (56.4%), and visual impairments (55.7%) were the most prevalent symptoms. The most common signs were nuchal rigidity (45.3%), altered mental status (26.9%), and papillary edema (37.5%). Brain computed tomography (CT) was normal in 68.6% of the patients while 22.9% of the cases had hydrocephalus. Brain magnetic resonance imaging (MRI) was normal in 60.0% of the patients. The most common abnormal MRI findings were leptomeningeal enhancement (16.0%) and hydrocephalus (16.0%). We had a 44.3% definite diagnosis with bacterial (n:25, 31.6%) and neoplastic (n:8, 10.1%) being the most prevalent etiologies. *Mycobacterium tuberculosis* (60%) and *Brucella* spp. (12%) were the most prevalent bacterial pathogens.

**Conclusions:**

The most common etiologies include infectious, neoplastic, and immunologic. Due to insidious presentation and uncommon etiologies, establishing a proper diagnosis, and providing timely targeted treatment for patients with subacute and chronic meningitis remains a challenge for clinicians.

## Introduction

Meningitis, a major neurological emergency, is inflammation of any of the three meningeal layers. Meningitis is classified into acute, subacute, and chronic meningitis based on symptoms duration of ≤ 5 days, ≥ 5 days and ≥ 4 weeks, respectively. Chronic meningitis occurs rarely and accounts for almost 10% of all meningitis patients [1–4]. To date, several etiologies have been identified for meningitis. The etiologies differ in acute meningitis compared to subacute and chronic meningitis. Community-acquired bacteria and viruses tend to cause acute meningitis, whereas atypical indolent bacteria (mainly *Mycobacterium (M.) tuberculosis*), fungi (predominantly *Cryptococcus neoformans*), and non-infectious etiologies cause subacute and chronic meningitis [4–7]. Subacute meningitis has less bacterial etiologies and more fungal causes compared to acute meningitis. Also, subacute, and chronic meningitis present with lower cerebrospinal fluid (CSF) white blood cells (WBC) count and higher rate of decreased CSF glucose in comparison with acute meningitis. Chronic meningitis tends to have milder symptoms compared to acute meningitis [4, 7].

Characteristics of the immune system, socioeconomic status, location, travel history, medical history, and medications are factors that influence the prevalence and the emergence of symptoms [8, 9]. The patient’s history and physical examination are of most value in the diagnosis of chronic meningitis. The most typical symptoms are headache, nausea, vomiting, and cranial nerve neuropathies. Other factors affecting the patient, including past medical history, medication history, intravenous drug use, sexual history, and recent travel history, must be considered when compiling a medical history. Regarding physical examination, we have to search for any skin lesions, ophthalmologic signs, signs of pulmonary infections. A thorough and precise neurologic examination is required [10, 11]. The next step is to perform radiology tests and CSF analysis for CSF opening pressure, glucose level, WBC count, and protein level. The most prevalent findings of CSF analysis are lymphocytic pleocytosis, elevated protein level, and normal or decreased glucose. Ventriculitis, diffuse leptomeningeal enhancement, hydrocephalous, and cranial nerve enhancement are significant findings on magnetic resonance imaging (MRI) with gadolinium contrast [6, 12]. Finally, when a definitive diagnosis is not possible, a biopsy is performed to confirm the diagnosis [13]. Aside from the evaluations and diagnostic tests, teamwork, and collaboration between specialists in infectious disease, neurology, radiology, ophthalmology, and rheumatology is essential to reach an appropriate conclusion [10].

Accurate and timely diagnosis of chronic meningitis and its etiologies have remained a challenge because of broad range of etiologies, including infectious, autoimmune, and neoplastic diseases, which are difficult to differentiate [14]. In addition, the underuse of available diagnostic tests such as arboviral testing and polymerase chain reaction (PCR) for viral etiologies, as well as the limited sensitivity of CSF cultures, have made it more difficult to determine the etiology of meningitis [7].

Data regarding subacute and chronic meningitis are mainly derived from different case reports and case series. Moreover, the features have changed over time. Therefore, to elucidate the distinct features, etiologies, and trends, gathering more data from various databases and referral centers seems essential. Considering the mysterious course, indefinite paraclinical findings, and diverse etiologies of chronic meningitis, we aim to study subacute and chronic meningitis patients.

## Methods

### Study design

We searched the medical records database for the final diagnosis by “unspecified meningitis” and “chronic meningitis” keywords. Initially, 2,050 records were retrieved, and 1971 records were excluded after the screening. We have included the patients referred to three tertiary centers from June 2011 to June 2021 with a final diagnosis of “chronic meningitis” and patients with a final diagnosis of “unspecified meningitis” that had pleocytosis and symptom duration of more than five days in this study. Patients with acute meningitis (thunderclap headache and symptoms less than or equal to 5 days) were not evaluated in this article.

### Participants

Patients were classified based on symptom duration as chronic meningitis (symptoms lasting more than 4 weeks) and subacute meningitis (symptoms lasting more than 5 days).

### Variables

Patients’ information including age, gender, and duration of symptoms, symptoms (subjectively reported by the patient or family member or caregiver), signs, laboratory data, radiology tests, treatment regimes, definite diagnosis, and clinical outcome were collected from medical documents. Headache, fever, nausea and vomiting, visual impairments, seizure, neuropathies, night sweats, weight loss, dyspnea, cough, and tinnitus were presented symptoms. Altered mental status, nuchal rigidity, and papillary edema were important recorded signs. Regarding CSF analysis, pleocytosis was defined as the presence of more than 5 WBC/mm^3^ under microscopic examination. CSF protein above 50 mg/dl was considered as increased CSF protein. Decreased CSF glucose levels were defined as CSF glucose levels less than 0.5 simultaneous serum glucose level. Bacterial meningitis was confirmed by CSF-positive cultures. In the case of brucella meningitis, CSF wright test confirmed the diagnosis. Diagnosis of tuberculous meningitis (TBM) was confirmed by positive CSF *M. tuberculosis* complex culture and or PCR. Cases with negative evaluations were grouped as no definite diagnosis. Death was considered as mortality during the hospitalization.

### Ethics

Research Medical Ethics Committee reviewed and approved this study (approval number: IR.SBMU.RETECH.REC.1400.196). This study was conducted in accordance with the Declaration of Helsinki and institutional ethics guidelines. For the use of clinical data, written informed consent was obtained from the patients or their guardians.

### Statistical methods

All statistical analyses were carried out by SPSS (version 26.0; IBM, Armonk, NY, USA). Descriptive statistics were calculated for all demographic variables. Quantitative variables and Categorical variables were compared between etiology groups (bacterial, tuberculosis (TB), neoplastic, and no definite diagnosis), using Kruskal–Wallis test and Fisher’s exact test, respectively. To compare Quantitative variables between subacute and chronic groups, we used Mann–Whitney U test, and for categorical variables, Chi-square, or Fisher’s exact test. A *p*-value less than 0.05 was considered significant.

## Results

The study included 79 patients with a mean age of 37.3 years (range 6–95 years, median 33.0 years). Of these, 53 (67.1%) were male and 26 (32.9%) were female. The median duration of symptoms before hospitalization was 32.7 days (range 6–360 days, median 15.0 days). According to the duration of symptoms, the number of cases with subacute meningitis and chronic meningitis was 32 (45.7%) and 38 (54.3%), respectively.

The most common symptoms were headache (87.3%), nausea and vomiting (74.7%), fever (56.4%), and visual impairments (55.7%). The most common signs were nuchal rigidity (45.3%), altered mental status (26.9%), and papillary edema (37.5%). Brain CT was normal in 68.6% of patients, while hydrocephalus was found in 22.9% of cases. Brain MRI was normal in 60.0% of the patients. The most common abnormal MRI findings were leptomeningeal enhancement (16.0%) and hydrocephalus (16.0%). Patients’ characteristics were summarized in Table 1.

**Table 1:** Detailed patients' characteristics compared between etiology groups

Characteristics	**Patients, No. (%)**							
	Total (*n* = 79)	Autoimmune (*n* = 1)	Viral (*n* = 1)	Bacterial, excluding TB (*n* = 10)	TB (*n* = 15)	Neoplastic (*n* = 8)	No definite diagnosis (*n* = 44)	*P*-value**‡**
Age, mean, years	37.3	39	18	35.1	29.7	57.4	37.2	**0.00**
Gender								
male	53/79 (67.1)	1(100)	1 (100)	7 (70.0)	8 (53.3)	2 (25.0)	34 (77.3)	**0.02**
female	26/79 (32.9)	0	0	3 (30.0)	7 (46.7)	6 (75.0)	10 (22.7)	
Symptoms								
Duration, mean, days	32.7	30	6	59.3	33.5	31.1	25.9	0.70
Headache	69/79 (87.3)	1(100)	1 (100)	10 (100)	14 (93.3)	3 (37.5)	40 (90.9)	**0.00**
Fever	44/78 (56.4)	0	1 (100)	5 (50.0)	15 (100)	1 (12.5)	22 (51.2)	**0.00**
Nausea/vomiting	59/79 (74.7)	0	1 (100)	7 (70.0)	12 (80.0)	5 (62.5)	34 (77.3)	0.75
Neurological symptoms^a^	31/79 (39.2)	0	0	3 (30.0)	9 (60.0)	6 (75.0)	13 (29.5)	**0.03**
Visual impairment								
Blurred vision	23/79 (29.1)	1(100)	1 (100)	4 (40.0)	3 (20.0)	3 (37.5)	11 (25.0)	0.41
Diplopia	18/79 (22.8)	0	0	4 (40.0)	4 (26.7)	3 (37.5)	7 (15.9)	
Photophobia	3/79 (3.8)	0	0	0	1 (6.7)	0	2 (4.5)	
Seizure	11/72 (15.3)	0	0	1 (11.1)	3 (20.0)	1 (12.5)	6 (15.8)	0.96
Night sweats	5/79 (6.4)	0	0	0	2 (13.3)	0	3 (7.0)	0.72
Weight loss	6/79 (7.6)	0	0	1 (10.0)	2 (13.3)	1 (12.5)	2 (4.5)	0.38
Dyspnea	2/79 (2.5)	0	0	0	0	0	2 (4.5)	1.00
Cough	5/79 (6.3)	0	0	0	1 (6.7)	1 (12.5)	3 (6.8)	0.80
Tinnitus	1/79 (1.3)	0	0	0	0	0	1 (2.3)	1.00
Medical history								
Immunosuppression^b^	2/79 (2.5)	0	0	1	1	0	0	-
Malignancy	8/79 (10.1)	0	0	0	0	6	2	-
Signs								
Altered mental status	21/78 (26.9)	0	0	1 (11.1)	8 (53.3)	3 (37.5)	9 (20.5)	0.57
Nuchal rigidity	24/53 (45.3)	0	0	6 (75.0)	8 (72.7)	0	10 (34.5)	**0.01**
Papillary edema	9/24 (37.5)	1(100)	0	2 (20.0)	0	1 (12.5)	5 (11.4)	**0.02**
Laboratory analysis								
WBC count, mean, × 10^9^/L	9.8	11.2	8.5	13.9	10.4	6.2	9.1	**0.02**
CSF analysis								
WBC count, mean, cells/mm^3^	1248.3	40	740	8199.4	201.6	334.5	380.3	0.50
Pleocytosis								
Neutrophilic	24/62 (38.7)	1(100)	0	6 (100)	2 (15.4)	1 (25.0)	14 (37.8)	**0.04**
Monocytic	34/62 (54.8)	0	1 (100)	0	10 (76.9)	3 (75.0)	20 (54.1)	
Lymphocytic	4/62 (6.5)	0	0	0	1 (7.7)	0	3 (8.1)	
Elevated Protein	49/67 (73.1)	0	1 (100)	6 (75.0)	11 (91.7)	4 (100)	27 (65.9)	0.24
Decreased glucose	47/54 (87.0)	1(100)	0	5 (83.3)	11 (100)	3 (75)	27 (87.1)	0.43
Radiology								
Normal Brain CT scan	24/35 (68.6)	-	-	3 (75)	6 (60)	1 (50)	14 (73.7)	0.75
Normal Brain MRI	15/25 (60.0)	0	-	2 (100)	2 (33.3)	1 (33.3)	10 (76.9)	**0.02**
Management								
Steroid therapy	30/79 (38.0)	1(100)	1 (100)	2 (20)	6 (40)	7 (87.5)	14 (31.8)	0.06
Anti-TB therapy	25/77 (32.5)	0	0	0	15 (100)	0	11 (25.6)	**0.00**
Empirical acyclovir therapy	23/64 (35.9)	-	1 (100)	3 (33.3)	7 (46.7)	2 (40)	10 (29.4)	0.53
Empirical antibiotic therapy	55/60 (91.7)	-	0	8 (100)	10 (90.9)	3 (60)	34 (97.1)	**0.04**
Death	**12/75 (16.0)**	**0**	**0**	**1 (11.1)**	**4 (26.7)**	**1 (16.7)**	**6 (14.0)**	**0.65**

*TB* Tuberculosis, *WBC* White blood cells, *CSF* Cerebrospinal fluid, *CT* Computed tomography, *MRI* Magnetic resonance imaging.

^a^ Focal neurologic deficits and altered level and content of consciousness.

^b^ History of HIV (human immunodeficiency virus) or immunosuppressant medications.

‡ The comparisons have been made among etiology groups (bacterial, TB, neoplastic, and no definite diagnosis.)

Regarding etiology, there was no significant difference between subacute and chronic meningitis (*p*-value = 0.56). Comparison of other clinical features is showed in Table 2.

**Table 2:** Detailed patients' characteristics compared between subacute and chronic meningitis

**Characteristics**	**Patients, No. (%)**		
	Subacute (*n* = 32)	Chronic (*n* = 47)	*P*-value
**Age,** mean, years	34.2	39.4	0.13
**Gender**			
**Male**	23/32 (71.9)	30/47 (63.8)	0.48
**Female**	9/32 (28.1)	17/47 (36.2)	
**Symptoms**			
**Headache**	29/32 (90.6)	40/47 (85.1)	0.73
**Fever**	24/32 (75.0)	20/46 (43.5)	**0.01**
**Nausea/Vomiting**	29/32 (90.6)	30/47 (63.8)	**0.01**
**Neurological symptoms**^a^	7/32 (21.9)	24/47 (51.1)	**0.01**
**Visual impairment**			
**Blurred vision**	10/32 (31.3)	13 (27.7)	0.29
**Diplopia**	4/32 (12.5)	14/47 (29.8)	
**Photophobia**	1/32 (3.1)	2/47 (4.3)	
**Weight loss**	1/32 (3.1)	5/47 (10.6)	0.39
**Night sweats**	1/31 (3.2)	4/47 (8.5)	0.64
**Seizure**	5/30 (16.7)	6/42 (14.3)	1.00
**Dyspnea**	1/32 (3.1)	1/47 (2.1)	1.00
**Cough**	1/32 (3.1)	4/47 (8.5)	0.64
**Tinnitus**	1/32 (3.1)	0/47	0.40
**Signs**			
**Altered mental status**	10/32 (31.3)	11/46 (23.9)	
**Nuchal rigidity**	13/24 (54.2)	11/29 (37.9)	0.27
**Papillary edema**	2/10 (20.0)	7/14 (50.0)	0.21
**Laboratory analysis**			
**WBC count,** mean, × 10^9^/L	10.8	9.0	0.18
**CSF analysis**			
**WBC count,** mean, cells/mm^3^	2244.3	482.1	**0.04**
**Pleocytosis**			
**Neutrophilic**	7/29 (24.1)	17/33 (51.5)	**0.00**
**Monocytic**	22/29 (75.9)	12/33 (36.4)	
**Lymphocytic**	0/29	4/33 (12.1)	
**Elevated protein**	23/29 (79.3)	26/38 (68.4)	0.40
**Decreased glucose**	25/27 (92.6)	22/27 (81.5)	0.42
**Radiology**			
**Normal brain CT scan**	13/17 (76.5)	11/18 (61.1)	0.39
**Normal brain MRI**	6/8 (75.0)	9/17 (52.9)	0.67
**Management**			
**Steroid therapy**	14/32 (43.8)	17/35 (48.6)	0.80
**Anti-TB therapy**	13/32 (40.6)	13/45 (28.9)	0.33
**Empirical acyclovir therapy**	11/31 (35.5)	12/33 (36.4)	1.00
**Empirical antibiotic therapy**	28/30 (93.3)	27/30 (90.0)	1.00
**Death**	**7/31 (22.6)**	**5/44 (11.4)**	**0.22**

*WBC* White blood cells, *CSF* Cerebrospinal fluid, *CT* Computed tomography, *MRI* Magnetic resonance imaging, *TB* Tuberculosis

^a^ Focal neurologic deficits and altered level and content of consciousness

Thirty-five cases (44.3%) were diagnosed with definite bacterial (n: 25, 31.6%), viral (n: 1, 1.3%), neoplastic (n: 8, 10.1%) and autoimmune (n: 1, 1.3%) meningitis. *M. tuberculosis* (60%) and *Brucella* spp. (12%) were the most prevalent bacterial pathogens. The identified etiologies of viral and autoimmune meningitis were varicella zoster virus (VZV) and sarcoidosis, respectively. Detailed bacterial and neoplastic etiologies are summarized in Table 3.

**Table 3:** Detailed etiologies of neoplastic and bacterial causes

Etiologies	Patients, No
Neoplastic (*n* = 8)	
B-cell lymphoma	2
Breast cancer	2
Lung cancer	1
CLL	1
Unknown origin	2
Bacterial excluding tuberculosis (*n* = 10)	
*Brucella* spp.	3
*Streptococcus pneumoniae*	2
*Neisseria meningitidis*	2
*Listeria monocytogenes*	2
*Acinetobacter baumannii*	1

*CLL* Chronic lymphocytic leukemia

All the TBM patients received standard first-line anti-TB. For patients with *Streptococcus pneumoniae* vancomycin and ceftriaxone were administered. *Neisseria meningitidis* was treated with Ceftriaxone. For patients with *Listeria monocytogenes* ampicillin was continued after initial empiric combined regimen. *Acinetobacter baumannii* was treated with meropenem and colistin. *Brucella* spp. was treated with ceftriaxone, doxycycline, and rifampin.

Of patients with no definite diagnosis, 17 (38.6%) cases were screened for neurosyphilis, and 19 (42.3%) patients were investigated for neurobrucellosis. Cryptococcal meningitis was examined in 16 (36.4%) patients, either by Indian ink staining, fungal culture or cryptococcal antigen. Further investigations for patients with no definite diagnosis are described in Table 4.

**Table 4:** Diagnostic tests performed on patients with no definite diagnosis (*n* = 44)

Tests	Patients, No. (%)
Microbiology analysis	
Gram stain	15 (34.11)
Indian ink stain	1 (2.3)
Cultures	
Blood culture	42 (95.5)
CSF bacterial culture	42 (95.5)
CSF fungal culture	13 (29.5)
Serology tests	
VDRL or RPR	17 (38.6)
Wright or 2ME	19 (43.2)
Cryptococcal antigen	4 (9.0)
CSF cytology	4 (9.0)
CSF PCR tests	
TB	6 (13.6)
HSV	6 (13.6)
BK virus	2 (4.5)
CMV	3 (6.8)
Brain biopsy	2 (4.5)

*CSF* Cerebrospinal fluid, *VDRL* Venereal disease research laboratory test, *RPR* Rapid plasma reagain, *2ME* 2-Mercaptoethanol, *PCR* Polymerase chain reaction, *TB* Tuberculosis, *HSV* Herpes simplex virus, *CMV* Cytomegalovirus

Patients with neoplastic etiology were significantly older than other cases. Headache and fever were significantly less in neoplastic patients compared to other etiology groups. Neurological symptoms were also significantly higher in cases of neoplastic and TBM compared to bacterial and unknown meningitis. WBC counts were significantly higher in patients with bacterial meningitis than in patients with neoplastic and unknown meningitis.

## Discussion

We conducted a retrospective study on patients admitted due to chronic and subacute meningitis in three tertiary hospitals in the past ten years. Etiology, epidemiology, sign and symptoms, imaging findings, and treatment choices were recorded and evaluated. Symptoms were broad and vague, but headache, nausea and vomiting, fever, and visual impairments were the most prevalent symptoms. Nuchal rigidity, altered mental status, and papillary edema were the most common detected signs. In imaging modalities, hydrocephalus on CT scan and leptomeningeal enhancement and hydrocephalus on MRI were recorded in several patients, but it was normal in most cases. The sensitivity and specificity of CT scan and MRI in subacute and chronic meningitis were lower early in the course of the disease compared to acute meningitis and could vary based on the etiology and timeline of the disease period [15]. Based on the prevalence of etiology, patients can be separated into three major groups: infectious, neoplastic, and autoimmune type.

*M. tuberculosis* and *Brucella* spp. were common bacterial pathogens in our study. Headache, fever, nausea, and vomiting were the most common symptoms of patients with a bacterial pathogen. Fever and neurological manifestations were more predominant in TB patients than in other bacterial etiologies. Despite the high rate of neurological manifestation in patients with underlying malignancy, headache, nuchal rigidity, and leukocytosis were low. WBC in CSF analysis was low in both neoplastic and TB patients. Although the most commonly encountered opportunistic central nervous system (CNS) infections in AIDS patients are *Toxoplasma gondii, JC virus, Cryptococcus neoformans* and cytomegalovirus [16], as showed in the results, in AIDS patients with chronic or subacute meningitis, TB still should be considered, especially in developing countries. As Cherian et al. demonstrated, TB as an important infectious cause of chronic meningitis can often evade a definite diagnosis. The CSF analysis and neuroimaging may be negative, and test repetition is needed. Also, they suggest using GeneXpert/LPA and BACTEC to confirm the diagnosis. Although they declared that a negative result for these tests, does not rule out TB [17]. Therefore, empirical therapy with TB treatments and corticosteroids seems to be a reasonable choice in suspicious cases. In neurobrucellosis, another important cause of chronic meningitis, Dar et al. study showed CSF routine analysis and neuroimaging could be normal, and serology tests and tracing antibodies are needed to confirm the diagnosis [18].

Autoimmune diseases exert adverse effects on CNS and various organs. Neurological symptoms can be detected as early signs of autoimmune disease or later in the course of the disease. We found sarcoidosis as a potential cause of chronic meningitis. A 30-day headache and blurred vision were the patient’s symptoms, and further evaluation revealed papillary edema on physical examination and MRI showed leptomeningeal enhancement. Neurosarcoidosis (NS) occurs in 5–10% of patients previously diagnosed with sarcoidosis [19]. Although, NS prevalence may be higher due to patients with NS having no other typical signs of sarcoidosis. Chronic autoimmune meningitis is not limited to sarcoidosis. Jarius et al. showed CSF analysis in 35% of patients with neuromyelitis optica (NMO), a serum immunoglobulin G (IgG) antibody-related disease, matched with chronic meningitis criteria [20]. Furthermore, chronic meningitis has been reported as aseptic meningitis in systemic vasculitis. Although chronic meningitis has been noticed in patients with systemic lupus erythematosus or Behcet disease, it is difficult to diagnose chronic meningitis in patients with early onset of systemic vasculitis [11]. Isolated CNS vasculitis can cause chronic meningitis without systemic manifestation. Brain biopsy is the gold standard. However, pleocytosis and segmental narrowing in cerebral angiography are helpful guides and clues [21]. As mentioned, autoimmune diseases can cause chronic meningitis in various mechanisms, so steroid therapy could be a reasonable consideration in these patients.

More than half of the diagnosed patients were not confirmed but improved during initial empirical treatment. Insidious onset, non-specific symptoms and its various etiologies have made the diagnosis of chronic meningitis difficult. In our study, steroid therapy improved outcomes in patients without a definite diagnosis and clear etiology. Smith et al. declared that 30% of their patients with chronic meningitis did not have a definite diagnosis and steroid therapy was effective in 52% of the patients [22]. Corticosteroid therapy may be useful not only for chronic autoimmune meningitis but also for TBM, drug-induced chronic meningitis, IgG4-related diseases, and certain genetic diseases such as neonatal multisystem inflammatory disease and Fabry disease [11]. We believe that using various techniques such as next-generation sequencing or brain biopsy may decrease the number of unconfirmed diagnoses and specify uses of steroid and antimicrobial therapy; however, these methods are not readily available [14].

Different types of meningitis are on a spectrum. As Sulaiman et al. demonstrated, subacute meningitis tends to be higher in immunosuppressed patients than acute meningitis. Serum and CSF leukocyte count and CSF glucose were lower in subacute patients, but positive gram stain and fungal culture were higher. They declared that age > 65, neurological symptoms in both acute and subacute, and fever in acute meningitis are associated with poor outcomes [7]. Contrastingly, our study revealed higher CSF leukocyte count in subacute cases compared to chronic cases. On the other part of this spectrum, fever, nausea and vomiting, and neurological symptoms were significantly lower in chronic meningitis in our study. CSF analysis revealed neutrophilic pleocytosis in chronic and monocytic pleocytosis in subacute meningitis. No significant difference was detected in age, gender, and radiological findings.

Sometimes the boundaries between acute and chronic meningitis become indistinguishable. Infectious etiologies of acute meningitis may unexpectedly result in subacute or chronic meningitis. Some patients with subacute meningitis were infected by *Neisseria meningitidis,* VZV*, Listeria monocytogenes, Streptococcus pneumoniae*, and *Acinetobacter baumannii.* Lefevre et al. demonstrated that *Neisseria meningitidis* can result in chronic meningitis, which mainly presents with fever, rash, and arthralgia in young male patients [23]. Our patients had a shorter symptom duration prior to admission (seven days compared to 28 days) and did not have rash and arthralgia. Tabaja et al. showed VZV could cause both encephalitis and meningitis. Meningitis is more expected in younger populations [24]. Subacute and chronic listeriotic meningitis are thought to typically occur in immunocompromised patients, but as reported by Arslan et al., immunocompetent patients developed listeriosis meningitis with a course of more than seven days [25].

Mandal et al. declared that the preceding use of antibiotics could affect the CSF analysis results. However, they believed this limited use of some oral agents did not significantly impact CSF analysis and diagnostic approaches. [26]. A history of two weeks of antibiotic use for otitis media prior to admission, and a history of corticosteroid use were noticed in the two patients with *Streptococcus pneumoniae* subacute meningitis. Antibiotics did not affect the results of CSF culture, but they may be the cause of uncommon presentation of a typical acute meningitis etiology. Moreover, in this patient the symptoms could be attributed to severe acute otitis media instead of meningitis. Thus, common acute meningitis etiologies can cause subacute and chronic meningitis due to previous use of antibiotics or corticosteroids.

Based on this study, we suggest an individual approach for diagnosing and treating chronic meningitis. TBM rate is high in particular areas, especially in low-income patients, while the neoplastic type was higher in other areas. Therefore, geographic epidemiology and socioeconomic situation should be considered in the differential diagnosis of chronic meningitis. Etiologic pathogens of acute meningitis can cause subacute and chronic meningitis in some patients. Considering the retrospective nature of our study, we weren’t able to homogenize the diagnosis approach for patients, and some procedures, such as brain biopsy, wasn’t performed for all undiagnosed patients. Also, the lack of a long-term follow-up limited the evaluation of sequelae.

## Conclusions

Chronic meningitis remains a challenging diagnosis even in a referral and experienced center. The most common etiologies include infectious, neoplastic, and immunologic causes. However, a significant number of cases had no definite diagnosis. To reduce the number of undiagnosed cases, complete and repeated evaluation of autoimmune etiologies and meningeal biopsies could be helpful. In addition, the empiric treatment (anti-tuberculosis, anti-endemic fungi, or corticosteroids) could vary locally based on the prevalence of distinct etiologies.

## Data Availability

The data that support the findings of this study are available from Shahid Beheshti University of Medical Sciences, but restrictions apply to the availability of these data, which were used under license for the current study, and so are not publicly available. Data are however available from the corresponding author upon reasonable request and with permission of Shahid Beheshti University of Medical Sciences.
